# Changes in Cardiac Metabolism in Prediabetes

**DOI:** 10.3390/biom11111680

**Published:** 2021-11-12

**Authors:** Vera H. W. de Wit-Verheggen, Tineke van de Weijer

**Affiliations:** 1 Department of Nutrition and Movement Sciences, School for Nutrition and Translational Research in Metabolism, Maastricht University Medical Center, 6200 MD Maastricht, The Netherlands; vhw.de.wit@outlook.com; 2 Department of Radiology and Nuclear Medicine, Maastricht University Medical Center, 6200 MD Maastricht, The Netherlands

**Keywords:** cardiac metabolism, prediabetes, mitochondrial function, cardiac function

## Abstract

In type 2 diabetes mellitus (T2DM), there is an increased prevalence of cardiovascular disease (CVD), even when corrected for atherosclerosis and other CVD risk factors. Diastolic dysfunction is one of the early changes in cardiac function that precedes the onset of cardiac failure, and it occurs already in the prediabetic state. It is clear that these changes are closely linked to alterations in cardiac metabolism; however, the exact etiology is unknown. In this narrative review, we provide an overview of the early cardiac changes in fatty acid and glucose metabolism in prediabetes and its consequences on cardiac function. A better understanding of the relationship between metabolism, mitochondrial function, and cardiac function will lead to insights into the etiology of the declined cardiac function in prediabetes.

## 1. Introduction

Prediabetes, defined as impaired fasting glucose (fasting plasma glucose between 6.1 and 6.9 mmol/L) or impaired glucose tolerance (2-h plasma glucose between 7.8 and 11.0 mmol/L) [1], places individuals at high risk of developing type 2 diabetes mellitus (T2DM) and its cardiovascular disease (CVD)-related complications [2,3]. The increased risk of CVD is proportional to the fasting blood glucose in prediabetes [2,4,5] and is mainly caused by atherosclerosis, induced by the many risk factors that are characteristic for prediabetic patients, for instance, dyslipidemia and hypertension [6,7,8,9,10,11,12]. Atherosclerosis leading to ischemic heart disease has been extensively discussed in previous literature [13]. However, even when corrected for atherosclerosis, cholesterol values, bodyweight, blood pressure, and age, patients with prediabetes remain at increased risk for the development of heart failure, mainly through the development of diastolic dysfunction (in T2DM known as diabetic cardiomyopathy (DCM)) [14,15]. This phenomenon is also part of the spectrum better known as heart failure with a preserved ejection fraction (HFpEF) [16].

Interestingly, diastolic dysfunction has been shown to be present not only in T2DM but also in prediabetes [17]. Evidence associates higher glucose levels with lower cardiac function parameters in prediabetes [17], indicating that changes in cardiac function arise early in the development of T2DM. Changes in cardiac metabolism in response to hyperglycemia are considered to be an important pathway through which T2DM causes DCM [18,19], and this may already be at play in prediabetes. Recognition of these metabolic changes may help to better understand the underlying etiology of diastolic dysfunction in prediabetes, which provides a window of opportunity for the prevention of DCM in the early development of T2DM. This narrative review will, therefore, only focus on the possible cardiac metabolic mechanisms behind the declined cardiac function in prediabetes (irrespective of their CVD risk profile), as these changes precede the onset of DCM. We will not discuss other possible pathways such as oxidative stress and inflammation, nor DCM [20,21,22], which have already been extensively discussed in the literature.

## 2. Cardiac Fat

When energy intake exceeds expenditure, it eventually results in body fat accumulation, as can be seen in obesity [23]. The large surplus of nutrients also leads to the development of fat deposits in organs other than adipose tissue, such as skeletal muscle, the liver, and the heart. Such ectopic lipid accumulation has been related to insulin resistance in many tissues. Thus, cardiac fat accumulation may play an important role in the development of diastolic changes in the prediabetic heart. Cardiac lipid content can be studied by both an in vivo and an ex vivo approach.

In vivo studies use magnetic resonance spectroscopy (MRS) for the relative quantification of metabolites involved in lipid metabolism. Proton MRS (^1^H-MRS) generates a spectrum wherein multiple lipid signals (CH_2_ and CH_3_), a creatine (Cr) signal, and a water (H_2_O) signal can be distinguished. CH_2_/H_2_O is generally used as a parameter that reflects myocardial triglyceride content, and this mainly represents neutral lipid storage as triglycerides in the myocardium. With this in vivo technique, it was shown that the myocardial triglyceride content is increased in overweight and obese individuals [24] and possibly even more in prediabetes and T2DM [25], in comparison to lean individuals. In addition, myocardial triglyceride content was weakly associated with insulin sensitivity, as determined by the homeostasis model assessment index [25].

Ex vivo studies found increased intramyocardial lipid deposition in patients with metabolic syndrome (average HOMA score 4.2 ± standard deviation 0.5, which often is considered as prediabetes) [26], obesity or T2DM [27], compared to lean patients. In addition, Anderson et al. showed that in nondiabetic and diabetic individuals, the cardiac fat content correlated positively with the HbA1c [28]. These ex vivo studies suggest that cardiac fat already in prediabetic individuals is increased compared to healthy lean individuals [26,27,28], which is in line with the above-mentioned in vivo results of McGavock et al. [25].

Interestingly, several links between cardiac fat accumulation and cardiac function have been described. Van der Meer et al. showed that in lean individuals with a normal glucose metabolism (NGM), an increased myocardial triglyceride content (following a very low-calorie diet) measured by ^1^H-MRS was correlated with a decrease in diastolic function [29]. In addition, in individuals with metabolic syndrome (and a high HOMA score), a correlation was found between the amount of cardiac fat accumulation and the progression of cardiac dysfunction (measured by myocardial performance index and ejection fraction) [26]. The increased myocardial triglyceride content in overweight and obese individuals was accompanied by elevated LV mass and suppressed septal wall thickening as measured by cardiac imaging, compared to lean individuals [24]. This suggests that possibly in prediabetes, an increased cardiac fat storage may influence cardiac function negatively.

## 3. Adipose Tissue Surrounding the Heart

The fat deposits around the heart (epicardial adipose tissue and pericardial adipose tissue) also typically increase with overweight/obesity and are reported to be more pronounced in diabetic patients. These depots have not been measured specifically in prediabetic populations, and although specific data of these separate depots in prediabetes are lacking, it might be expected that the epicardial adipose tissue (EAT) is elevated in prediabetes since cardiac fat is increased in prediabetes and the thickness of the EAT is strongly correlated with the cardiac fat in healthy males [30]. However, quantitative studies are needed to confirm this concept in prediabetic individuals.

From obese individuals, it is known that when EAT expands, the balance between the storage and release of fatty acids shifts toward a more active secretion [31]. Furthermore, the expanded EAT transforms its secretory profile toward more pro-inflammatory cytokines and chemokines, negatively affecting neighboring cells [32,33,34]. This results in a chronic inflammatory response that is shown to be present in enlarged EAT tissue [35,36]. Moreover, this local secretion of inflammatory mediators can also inhibit the activity of insulin. Indeed, EAT is positively associated with insulin resistance and metabolic syndrome [37,38].

Literature shows that the expansion of EAT has a negative influence on cardiac function [39,40,41]. Although studies on EAT are lacking in prediabetes, this unfavorable effect of increased EAT on function parameters seems to be a general phenomenon and was reported in lean, obese, and T2DM individuals. First of all, pericardial fat thickness, measured from the long axis view, is shown to be a predictor of the mobility of the lateral left ventricle wall, known as e’ lateral [39]. Secondly, the thickness of EAT in morbidly obese individuals is associated with enlarged atria and impaired diastolic filling of the right and left ventricle [42]. This is in line with the findings in a healthy population with, on average, a normal BMI but with a high prevalence of the metabolic syndrome and T2DM, wherein PF volume was correlated with the left atrial diameter and with E/e’ [41]. In morbidly obese female individuals, the adipose tissue volume around the left ventricle did not only correlate with diastolic function parameters (peak early filling velocity (E) and peak late filling velocity (A)), but also with several left ventricular hemodynamic measurements, including cardiac output and stroke volume [40]. Furthermore, EAT is associated with left ventricular mass (LVM), which is a strong predictor of adverse cardiovascular outcomes [31,41].

The association between EAT and cardiac function may be explained by several mechanisms. Firstly, EAT is a storage depot for FFA and may thus provide the heart with nutrients [32], therewith contributing to the changed cardiac lipid metabolism. Secondly, the chronic inflammatory response, which is shown to be present in enlarged EAT tissue [35,36], and the inflammatory cytokines produced by EAT may act locally as paracrine atherogenic factors [32]. Finally, mechanistic hindrance may limit the distensibility of the myocardium [43].

As studies on the relationship of EAT with changes in cardiac metabolism and function are lacking in prediabetes, more research is warranted. Especially since it is known that EAT is more flexible and reduces even before the cardiac fat decreases [44]. Possibly, EAT contributes to diastolic dysfunction in prediabetes; however, to what extent remains to be elucidated.

## 4. Enhanced Cardiac Lipid Metabolism

Insulin usually inhibits lipolysis and thereby reduces the release of plasma non-esterified fatty acids (NEFAs). However, in individuals with reduced insulin sensitivity, as is the case in prediabetes, the postprandial effect of insulin is impaired, leaving the circulating free fatty acids (FFA) elevated [45]. In addition to the increased FFA levels in the circulation, PET studies show that both the FFA uptake and the FFA oxidation in the prediabetic myocardium are increased. Using ^18^F-fluoro-6-thia-heptadecanoic acid (FTHA) as a fatty acid tracer and [^11^C]acetate to determine cardiac perfusion and oxidative metabolic index, Labbé et al. showed that in prediabetic individuals (defined as impaired glucose tolerance), an increased NEFA uptake in the heart and an increased myocardial oxidative metabolism for the first 6 h postprandially compared to the individuals with a normal glucose metabolism (NGM) [46]. This was in contrast to the uptake of fatty acids in liver and skeletal muscle since these remained similar in prediabetes compared to NGM in the postprandial state [46]. These findings concerning increased FFA availability in the plasma and myocardial FFA metabolism are confirmed by Brassard et al. in normoglycemic first-degree relatives of T2DM individuals (who are therefore at highly increased risk to develop T2DM) in comparison to matched individuals having no increased risk for T2DM. Using the stable isotopic tracers ([1,1,2,3,3-^2^H_5_]-glycerol and [U-^13^C]-palmitate or [1,2-^13^C]-acetate), they showed that these individuals at high risk for T2DM during enhanced intravascular TG lipolysis at high insulin levels have both an increased plasma appearance of NEFAs and increased myocardial oxidation of the NEFAs [45].

These findings in the insulin-stimulated condition from Brassard and Labbé point out that already in prediabetes, changes in cardiac fatty acid handling occur, with increased uptake and oxidation of fatty acids in the heart in comparison to NGM. Moreover, Labbé et al. revealed that these changes in lipid metabolism may be maladaptive regarding cardiac function. The increased uptake and oxidation of NEFA in the prediabetic individuals was associated with a reduced left ventricular ejection fraction (LVEF), reduced left ventricular stroke volume, and tended to display impaired diastolic function [46]. This is in line with the findings from Mather et al. in T2DM individuals, who showed that the augmented myocardial fatty acid oxidation under fasted and insulin-treated conditions (measured by16-[^18^F]fluoro-4-thiapalmitate (FTP) and ^11^C-acetate) was accompanied by reduced cardiac work efficiency [47]. This may not be surprising since increased fatty acid oxidation at the expense of carbohydrate oxidation increases oxygen demand, resulting in reduced myocardial efficiency [47]. In addition, in prediabetic individuals with a known increased risk for atherosclerosis, this makes the heart more prone to ischemia. The enhanced fatty acid metabolism in prediabetes has, therefore, implications for contractile performance and ischemia tolerance [47].

It may be beneficial to counterbalance this altered substrate metabolism in order to prevent DCM in T2DM. The metabolic changes that occur early on in the prediabetic heart seem to be reversible, as shown by several studies in prediabetic individuals. Six months after bariatric surgery, individuals with prediabetes showed an improvement in whole-body insulin sensitivity, which correlated positively with the decrease in myocardial fasting free fatty acid uptake, but also myocardial function. Although cardiac fat was not reduced, the myocardial structure was improved [44]. Similar results in prediabetes were observed by Labbé et al., where modest weight loss following a 1-year lifestyle intervention led to changes in substrate metabolism and improved cardiac function [48]. However, a short-term diet of 7 days in prediabetes did not achieve these improvements in cardiac function [49] and thus suggesting that structural changes regarding cardiac metabolism and function take longer to develop.

## 5. Decreased Cardiac Glucose Metabolism

Together with alterations in cardiac fatty acid metabolism, reciprocal changes in cardiac glucose metabolism may be expected in prediabetes [50]. Here, PET studies using the glucose analog [^18^F]-fluorodeoxyglucose (^18^F-FDG) can provide insight into the myocardial uptake of glucose. Kim et al. studied a mixed population of NGM, prediabetes, and T2DM and revealed that the visceral fat area and fasting FFA are independent determinants of myocardial glucose uptake in the fasted condition [51]. However, both Kim et al. and Hu et al. showed that prediabetes was not associated with decreased myocardial glucose uptake in a fasted condition, whereas T2DM was [51,52], which is in line with animal studies [53]. However, findings might be different in a fed or insulin-stimulated state.

In contrast to the fasted individuals in the study of Kim et al., Nielsen et al. studied the myocardial glucose uptake 1 h after oral glucose intake in NGM, prediabetes, and newly diagnosed T2DM individuals, all characterized by chronic heart failure and reduced LVEF. Even though the myocardial blood flow and myocardial flow reserve were similar, individuals with prediabetes and newly diagnosed T2DM had-despite of elevated levels of glucose and insulin-a decreased myocardial glucose uptake compared to NGM [54]. However, since the authors did not separate analyses for individuals with prediabetes and T2DM, it is unknown whether there were differences between these groups.

To assess the insulin-stimulated myocardial glucose uptake in a more controlled setting than right after glucose ingestion, one should measure myocardial glucose uptake during a hyperinsulinemic euglycemic clamp [55]. Eriksson et al. showed a similar cardiac glucose metabolic rate during such clamp in control, prediabetes, and T2DM individuals matched for age, sex, and BMI [56]. Others showed lower myocardial glucose uptake in T2DM compared to (BMI-matched overweight) NGM individuals during a hyperinsulinemic euglycemic clamp [55,57]. These conflicting in vivo findings of myocardial glucose uptake in T2DM are also found in ex vivo studies. Full-thickness myocardial biopsies from the left ventricle of T2DM individuals showed an increase in cardiac insulin receptor substrate 1 (IRS1)–PI 3-kinases (PI3K) activity compared to their overweight controls with NGM [58]. This means that the insulin signaling cascade, even in this state of insulin resistance, is intact. However, differences between groups can be blunted due to the fact that all individuals in the ex vivo studies were characterized by left ventricular dysfunction.

Overall, results on myocardial glucose uptake in prediabetes are conflicting, both in in vivo and ex vivo studies. Some found no differences in healthy prediabetic individuals compared to NGM or T2DM individuals in a fasted state [51] and during a clamp [56], whereas others did find reduced myocardial glucose uptake 1 h after oral glucose loading [54] in prediabetic patients with chronic heart failure. In addition, previous literature is ambiguous whether myocardial glucose uptake is associated with whole-body insulin sensitivity [57] or not [56] measured during a hyperinsulinemic euglycemic clamp. Data are dispersed, and the question remains whether prediabetes is characterized by reduced myocardial insulin sensitivity.

The effect of altered myocardial glucose metabolism on cardiac function has, so far, only been studied in patients with heart failure. Animal studies show conflicting results. In diabetic Zucker rats, the decreased glucose use (assessed by ^18^F-FDG as PET tracer) was associated with impaired diastolic and systolic cardiac function (assessed upon ultrasound) [53]. Surprisingly, a study in insulin-resistant Sprague–Dawley rats showed an increased glucose use of the myocardium accompanied by a higher left ventricular ejection fraction, a smaller left ventricular end systolic volume, and a thicker end systolic wall thickness [59]. Hence, the mechanism remains unclear, and it is unexplored what the relation between glucose metabolism, insulin, and cardiac function is in prediabetes.

MRS studies focusing on the tracers hyperpolarized [1-^13^C]-pyruvate or [2-^13^C]-pyruvate provide mechanistical information and revealed defects in the carbohydrate metabolism on the level of PDH. Although only a few studies have been performed with this new technique, the first results are promising. Cunningham et al. showed that assessment of the cardiac pyruvate metabolism in vivo in humans is feasible [60], and Rider et al. showed a significantly reduced metabolic flux through cardiac pyruvate dehydrogenase in T2DM compared to their age-matched healthy controls [61]. Thus, in T2DM, in addition to insulin resistance, a reduced metabolic flux through pyruvate dehydrogenase can explain the decreased glucose uptake. In addition, a significant increase in metabolic flux through pyruvate dehydrogenase was observed in the T2DM individuals after the oral glucose loading [61]. The depressed flux through pyruvate dehydrogenase in T2DM individuals is in line with results from various animal models [62,63,64]. Chatham et al. found a depressed flux in both Zucker diabetic fatty rats [62] and in isolated perfused rat hearts with streptozotocin-induced diabetes [63]. Interestingly, PDH flux was associated with diastolic function [63,64]. Hopefully, future studies using this elegant method may provide more insight into the underlying mechanisms potentially modulating glucose and fat oxidation in the prediabetic state in humans.

In cases where changes in glucose metabolism were found in the prediabetic state, these were reversible, similarly to the possible alterations in lipid metabolism. Even within one month after bariatric surgery and subsequent weight loss, severely obese T2DM showed an increase in myocardial glucose uptake [65]. Hannukainen et al. studied 46 individuals with T2DM, impaired glucose tolerance, and NGM before bariatric surgery and six months after the surgically induced weight loss. Not only an improvement in whole-body insulin sensitivity was detected, which correlated positively with the increase in myocardial glucose uptake and the decrease in myocardial fasting free fatty acid uptake, also myocardial function, and myocardial structure were improved [44]. However, like the lifestyle intervention, which had positive effects on lipid metabolism, from this study, we neither know whether these changes are due to the whole-body effects of the weight loss. A 16-week intervention with the PPARγ-agonist rosiglitazone has proven to increase myocardial glucose uptake during a hyperinsulinemic euglycemic clamp in both ischemic and non-ischemic regions in individuals with T2DM and coronary artery disease [66], showing that myocardial glucose uptake can not only be affected by bariatric surgery or lifestyle adjustments but also by drugs.

## 6. Mitochondrial Function

Mitochondria are responsible for oxidative metabolism and are key to the normal function of the cardiomyocytes. It is, therefore, not surprising that mitochondrial dysfunction is suspected of playing a pivotal role in the development of DCM [67,68]. Unfortunately, human data in prediabetes in this area are lacking. Though studies in male Long-Evans rats that were high-fat-fed and had a streptozotocin treatment as a model for prediabetes have shown a mild diastolic dysfunction and cardiac hypertrophy associated with early changes in mitophagy [69]. This supports the suggestion that mitochondrial dysfunction underlies the development of DCM in prediabetes.

However, information from both in vivo and ex vivo studies performed in obesity and T2DM is available and may explain possible changes in mitochondrial function in prediabetes. From ex vivo studies using high-resolution respirometry as a reflection of mitochondrial function, it is known that lower mitochondrial respiration is associated with T2DM [28,70]. This indicates that the myocardium of T2DM individuals, in spite of their preference for fatty acid oxidation as shown in PET studies, has a decreased maximal capacity for fatty acid-supported respiration in comparison to nondiabetic individuals. Moreover, Anderson et al. reported a negative correlation of maximal capacity for fatty acid respiration with the HbA1c [28]. The relationship with Hb1A1c may suggest that mitochondrial function may already be affected in prediabetes.

In line with the notion of decreased mitochondrial function in metabolically challenged individuals such as prediabetes, some differences in mitochondrial function are already found between lean and obese individuals. Montaigne et al. found in vitro abnormal respiratory chain complex activities in obese individuals without T2DM, but this did not result in reduced mitochondrial respiration [70]. This is in line with Niemann et al., who showed that obese individuals had disturbed mitochondrial biogenesis and function (respiratory chain complex I) in the right atrial cardiomyocytes compared to lean individuals [71]. In addition, ex vivo contractile performance was decreased in obese individuals already before the onset of clinical cardiomyopathy, although to a lesser extent than in T2DM [70]. However, these results suggest that not only chronic hyperglycemia, as seen in T2DM, but already the early-stage alterations in glucose homeostasis as seen in obesity, have an impact on mitochondrial function and thereby on the intrinsic myocardial contractile function. It is, therefore, to be expected that these changes in obese individuals are also present in prediabetes as a prelude to DCM; however, this remains to be explored in new cross-sectional studies.

**Figure 1:**
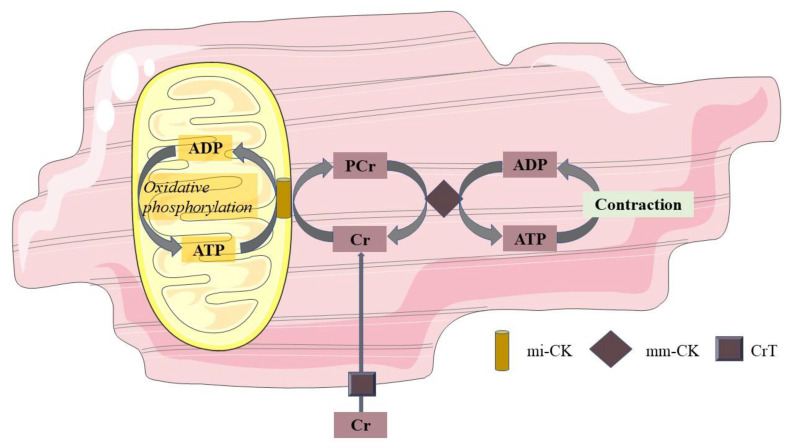
Phosphocreatine shuttle system. Mitochondria produce ATP during oxidative phosphorylation, and this ATP converts Cr at the mitochondrial membrane into PCr through mi-CK. PCr, in turn, shuttles from the mitochondrial membrane to the sarcolemma, where through the mm-CK, the phosphorous group of PCr is exchanged with ADP to form ATP in cases of increased energy demand. In this way, PCr acts to buffer ATP. ADP adenosine diphosphate; ATP adenosine triphosphate; Cr free creatinine; PCr phosphocreatinine; mi-CK mitochondrial creatine kinase; mm-CK myofibrillar creatine kinase; CrT creatinine transporter.

In vivo, concentrations of high-energy phosphates have been suggested to be closely related to cardiac mitochondrial oxidation. This can be measured by ^31^P-MRS. With this technique, PCr and ATP can be recognized at a specific resonance frequency. As a relative quantification, the ratio of PCr over ATP is used as a measure for myocardial energy status. Mitochondria produce ATP during oxidative phosphorylation, and ATP can, in turn, be used to convert creatine (Cr) into PCr. In the sarcolemma, the phosphate group of PCr is exchanged with adenosine diphosphate (ADP) to form ATP in case of increased energy demand. In this way, PCr acts to buffer ATP. This PCr shuttle system is also shown in Figure 1. In the normal myocardium, ATP synthesis can be maintained at the rate of ATP demand, and PCr levels are sustained. However, in cardiac disease with a decreased mitochondrial function, ATP demand may outweigh the mitochondrial capacity for ATP production, and hence, PCr concentrations will fall [72]. Hence, the PCr/ATP ratio has been suggested to be a marker of mitochondrial function; however, one should be aware that creatine supply, pH, and oxygen supply may independently influence PCr concentrations in the cardiomyocyte [73].

Since ex vivo studies hint toward a decreased mitochondrial function in prediabetes [28,70], it can be expected that in prediabetes, the myocardium may have to rely more on its reserves (PCr) for the production of ATP to meet the ATP demand, resulting in reduced myocardial energy status, as measured by PCr/ATP ratio in vivo. Despite that the literature on cardiac mitochondrial dysfunction in obesity and T2DM is expanding, studies in prediabetes are lacking. ^31^P-MRS in vivo studies performed by Diamant et al. and Scheuermann-Freestone et al. show in T2DM with a relatively high HbA1c (6.1 ± 1.1 and 8.3 ± 0.4, respectively) a lower PCr/ATP ratio compared to NGM, and are thus in line with the decreased mitochondrial function measured ex vivo [74,75]. However, in T2DM individuals with well-regulated plasma glucose, the PCr/ATP ratio found by Rijzewijk et al. was not different from matched obese controls [76]. In addition, Scheuermann-Freestone et al. showed that the PCr/ATP ratio correlated negatively with plasma FFA concentrations in T2DM and NGM and that PCr/ATP correlated positively with the plasma glucose concentrations in the individuals with T2DM, thus showing that metabolic dysregulation is a hallmark of a disturbed cardiac energy status [75] and thereby implying that this already could be the case in prediabetes.

Both in nondiabetic and in T2DM individuals, a lower PCr/ATP ratio is shown to be inversely associated with diastolic cardiac function parameters, such as, for instance, E acceleration peak, E deceleration peak, and E peak filling rate [74]. These human in vivo MRS data support the hypothesis that the early alterations in mitochondrial energy metabolism in the prediabetic state do increase the susceptibility to diastolic heart failure, as seen in DCM.

However, since in vivo studies in prediabetes are currently lacking, the expected decreased PCr/ATP ratio in prediabetes is a speculation based on data in obesity and T2DM. Therefore, the complex pathology of changes in mitochondrial function and metabolism in prediabetes remains incompletely understood. As already described in the section on cardiac fat, a potential mechanism may involve the detrimental effects of the excessive bioavailability of nutrients. Possibly these nutrients, or more specifically fatty acids, may influence mitochondrial function. In mouse studies, it has been shown that a high abundance of fatty acids may lead to inefficient substrate oxidation in the heart (reflected by a reduced ratio of energy production (ATP production) to respiration), resulting in the formation of reactive oxygen species (ROS) and thereby to mitochondrial damage [67,68,77,78]. Possibly, a similar mechanism occurs in the human heart in the prediabetic state.

**Figure 2:**
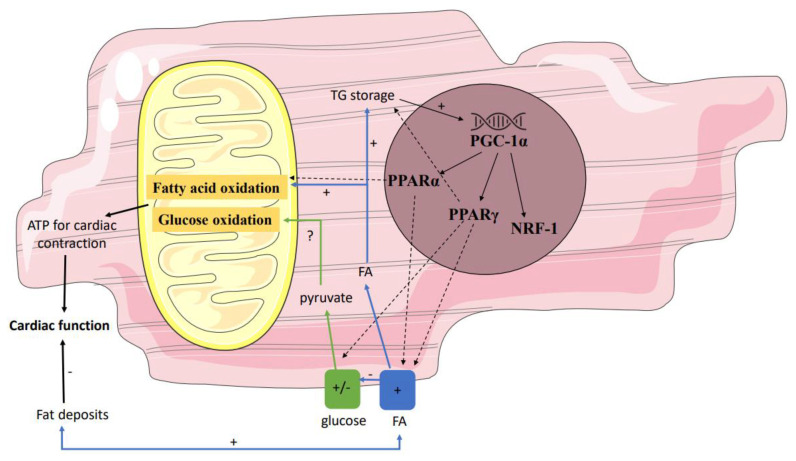
Gene regulatory pathways affecting mitochondrial function. Several genes influence the interplay between the supply and oxidation of the various substrates. Down- and/or upregulation of these genes in prediabetes may affect mitochondrial function. Partly because of this, in prediabetes fatty acid oxidation may be stimulated, resulting in a net reduction in ATP and thus reduced myocardial efficiency. PPAR peroxisome proliferator-activated receptor; ATP adenosine triphosphate.

Secondly, gene regulatory pathways may affect mitochondrial function by influencing the interplay between the supply and oxidation of the various substrates (Figure 2). Not only in individuals with T2DM but also in first-degree relatives of individuals with T2DM, it has been shown that the expression of peroxisome proliferator-activated receptor (PPAR) coactivator PGC-1α is decreased in skeletal muscle [79]. PGC-1 is usually increased upon cellular ATP demand [80], leading to transcription of NRF-1, PPARα, and PPARγ, and may thereby have indirect effects on mitochondrial metabolism. Since NRF-1 regulates the expression of many mitochondrial genes, including the OXPHOS genes, a decreased expression of PGC-1α will result in a lower mitochondrial content of the OXPHOS complexes. These data found in skeletal muscle may be translated to heart, though data in prediabetes are lacking. Yet, in T2DM, Montaigne et al. found a downregulation of NRF-1 and mitochondrial function in the heart [70].

In addition to the downregulation of NRF-1 following a decreased expression of PGC-1α, also PPARα and PPARγ can be influenced by NRF-1. These PPAR receptors are known to coordinate the expression of the most key regulators of the fatty acid metabolism and are thereby responsible for determining substrate preference in the heart. Polymorphisms within the human PPARα and PPARγ gene have been reported to influence risk markers for CVD, including BMI, cholesterol, and the incidence of T2DM [81]. Seen as PPAR polymorphisms are associated with the incidence of T2DM, a possible difference in PPAR expression in healthy compared to prediabetic and T2DM individuals may be expected. However, Marfella et al. studied biopsies of the left ventricular septum and did not find significant differences in myocardial PPARα expression between individuals with and without metabolic syndrome, although PPARγ was lower in the healthy individuals [26]. Moreover, the expression of PPARγ was correlated with LVEF and the accumulation of cardiac fat [26]. This is in line with the results of Anderson et al., who found a decreased mitochondrial respiration upon fatty acid stimulation but did not find differences in expression of PGC-1α nor PPARα in the heart atria in T2DM [28]. However, this was a relatively small and heterogeneous group of patients. Similarly, a large observational study in T2DM individuals (the FIELD study) showed that the use of a PPARα agonist (fenofibrate) did not reduce the risk of coronary events; however, it did reduce the total cardiovascular events, mainly due to fewer non-fatal myocardial infarctions and revascularizations [82], implicating that PPARα stimulation may have a beneficial effect in T2DM. The mechanism behind this advantageous effect in humans is unknown, but treatment with a PPARα agonist (GW7647) also showed in mice a protective effect on myocardial contractile function after induction of cardiac ischemia [83] and treatment with a PPARα agonist (fenofibrate or ciprofibrate) in different animal models of insulin resistance (high-fat diet induced in C57BL/6 mice or genetic induced in obese Zucker rats) showed a slight improvement of glucose metabolism [84]. The latter may possibly explain the beneficial effect of fenofibrate in the FIELD study if findings from animal studies can be translated to humans. On the contrary, other mouse studies showed that increased availability of fatty acids led to activation of the PPARα gene regulatory pathway, which resulted in an increased uptake of fatty acids and cardiac dysfunction in diabetic mice [85,86,87]. Thus, the effect of PPARα agonists on cardiac function is contradictory in different animal studies, and intervention studies in prediabetes and T2DM are not performed. Hence, the order of events remains unclear, and thus are studies in bigger populations are needed to pinpoint the relevance of PPAR gene regulatory pathways in the development of cardiac metabolic aberrations and cardiac dysfunction in prediabetes.

## 7. Conclusions

Data from clinical studies on cardiac metabolism in prediabetes are scarce. Myocardial triglyceride content is associated with insulin sensitivity [25] and is increased in prediabetes [25,26,27,28]. Although the different fat deposits around the heart have not been measured in prediabetic populations, it might be expected that the epicardial adipose tissue is elevated [30,37,38]. During insulin-stimulated conditions, both the FFA uptake and the FFA oxidation in the prediabetic myocardium are increased during insulin-stimulated conditions [45,46]. Although a vastly decreased glucose uptake and glucose oxidation have been shown in T2DM, the few studies in prediabetes show conflicting results, and the question remains whether prediabetes is characterized by a reduced myocardial insulin sensitivity [51,52,56,57]. Mitochondrial function is also not well studied in prediabetes, but it is likely that not only hyperglycemia as seen in T2DM, but already the early-stage alterations in glucose homeostasis as seen in obesity, have an impact on mitochondrial function as HbA1c is negatively correlated to maximal fatty acid respiratory capacity [28] and plasma glucose concentrations are correlated with PCr/ATP [75]. It is, therefore, to be expected that a decreased mitochondrial function is also present in prediabetes as a prelude to mitochondrial dysfunction in DCM [67,68].

The metabolic changes have consequences in prediabetic individuals, as metabolic influences on cardiac function are often seen in different patient populations. Cardiac function was negatively influenced in both healthy and metabolically compromised individuals by increased fat storage [24,26,29], increased epicardial adipose tissue [39,40,41], increased FFA uptake and oxidation [46,47], and a lower PCr/ATP ratio [74]. Although studies in prediabetic individuals are lacking, these results do support the notion that metabolic changes in prediabetes might contribute to the development of diastolic dysfunction, as seen in DCM.

The metabolic changes and the associated functional impairment in the prediabetic heart do seem to be reversible [44,48,65,66]. Hence, it seems to be important to counterbalance these changes in substrate metabolism in the early (pre)diabetic state and improve mitochondrial function, as these changes precede DCM in T2DM. This emphasizes the need for studies intervening in the prediabetic state to allow a better cardio-protection in the development of T2DM and the metabolic syndrome.

## Abbreviations

^1^H-MRS	proton MRS
^18^F-FDG	[^18^F]-fluorodeoxyglucose
^18^F-FTHA	^18^F-fluoro-6-thia-heptadecanoic acid
^18^F-FTP	16-[^18^F]fluoro-4-thiapalmitate
A	peak late filling velocity
ADP	adenosine diphosphate
ATP	adenosine triphosphate
CVD	cardiovascular disease
Cr	creatine
DAG	diacylglycerol
DCM	diabetic cardiomyopathy
E	peak early filling velocity
EAT	epicardial adipose tissue
FFA	free fatty acid
HSL	hormone-sensitive lipase
IRS1	insulin receptor substrate 1
LVEF	left ventricular ejection fraction
LVM	left ventricular mass
MRS	magnetic resonance spectroscopy
NEFA	nonesterified fatty acid
NGM	normal glucose metabolism
PCr	phosphocreatine
PET	positron emission tomography
PI3K	PI 3-kinases
PPAR	peroxisome proliferator-activated receptor
ROS	reactive oxygen species
T2DM	type 2 diabetes mellitus
TG	triglyceride
